# A Disaster Medical Assistance Team Operates a Hurricane Evacuation Shelter With U.S. Public Health Service Support

**Published:** 2006-03-15

**Authors:** Luis Rios, Theresa Cullen

**Affiliations:** Orange Park Medical Center, Department of Emergency Services, Orange Park, Fla; U.S. Public Health Service, Indian Health Service, Tucson, Ariz

Several types of federal health response teams operate in times of national disaster. Among these are disaster medical assistance teams (DMATs), which are assigned to emergency locations by the Federal Emergency Management Agency through its National Disaster Medical System (NDMS) (1). A DMAT consists of medical and nonmedical volunteers (usually from the same state or region of a state) who form a response team under the guidance of NDMS or under similar state or local auspices (1). A DMAT team typically includes 35 individuals, including physicians, nurses, nurse practitioners, physician's assistants, pharmacists, emergency medical technicians, other allied health professionals, and support staff (1), reflecting the professional and technical expertise of a small community hospital or a trauma unit.

Teams come equipped with their own medical supply cache and have the capability to set up a MASH-style tent in the field (2). A standard DMAT can be "dropped" into a disaster situation and may include a pharmacy, a laboratory for blood analysis, x-ray augmentation capability, resuscitation equipment, monitoring equipment, generators, pressurized tents, and refrigeration.

Another team example is the U.S. Public Health Service (USPHS) Commissioned Corps. These teams vary in size and responsibility and are assigned by the Department of Health and Human Services to federal, state, or local agencies or international organizations to accomplish their various missions, one of which is to furnish health expertise in times of emergency (3). A common USPHS task after a national disaster is to assess the needs of shelters, devastated communities, damaged hospitals, and extended patient care facilities. This letter reports the experience of using complementing DMAT and USPHS teams in a small city in central Texas beginning 48 hours after Hurricane Rita hit the Gulf Coast in September 2005. The goal of these two teams was to establish a shelter for evacuees from Hurricane Rita.

The initial assignment for our 35-member DMAT team was to provide services to a special-needs population requiring care and support for acute medical conditions. However, the needs were much broader than expected and included receiving and sheltering hurricane evacuees with various requirements: chronically ill individuals, individuals with acute and chronic conditions, members of the general population, and individuals from different communities and states. These people were transported from primary shelters in San Antonio, Port Arthur, Houston, and Galveston, Tex, as well as coastal areas of Louisiana. The medications of most evacuees were collected by nurses riding with them on buses hired by the state; this system later created confusion because the medications were returned to the shelter's treatment stations rather than to disembarking patients. No individual triage or needs assessment had been conducted before evacuees arrived at the shelter.

A first challenge was that the local organizers expected to provide command for the shelter. Local organizers included the emergency operation center, emergency medical services, a large family practice group, and a coalition of nonprofit agencies associated with the local emergency operation center. The six-member USPHS advance team, which included two physicians, three nurses, and an epidemiologist, arrived just before the DMAT and had already made decisions on sanitation and infection control. The teams and local stakeholders agreed that the shelter would exist as a multidisciplinary medical entity. All personnel attended daily briefings on shelter operations, and ultimately, the partnership provided command.

The next hurdle was the realization that the special needs–acute care paradigm was not accurate and that the evacuees would have to be sorted out in a quick and medically controlled way. We used the START (simple triage and rapid treatment) system (4) for the arrival of the first evacuees. Triage was performed by the most skilled medical and prehospital (i.e., emergency medical technicians and paramedics) personnel. We reorganized the way we received evacuees as we identified new needs. As a result, the reception process eventually addressed the evacuees' needs for privacy, quiet, sunshine, and socialization. At least two additional entrances and exits were added to fulfill these needs. The treatment stations for people with highly acute conditions were supported by the local emergency medical system, hospitals, and clinical specialists.

The shelter served approximately 450 men, women, and children. Approximately 40% of the evacuees did not have medical conditions that required attention. The remainder were screened and treated at the treatment stations; these individuals had health conditions such as oxygen-dependent pulmonary disease, complex congestive heart failure, asthma, acute myocardial infarction, ectopic and high-risk pregnancy, fractures, diabetes, and seizure disorder. Twelve evacuees were dependent on oxygen support, six morbidly obese evacuees required large air mattresses and skin care, and 12 evacuees with Alzheimer's disease required continuing attention. The DMAT behavioral health group treated 12 adults with bipolar disorder or schizophrenia and one autistic child; these individuals were housed in a secluded area away from distractions.

Two Veterans Administration (VA) hospitals had the potential to house and care for the majority of the evacuees, but these hospitals required some repair. The USPHS team assessed these sites, the needs of the evacuees were matched to the capabilities of each hospital, and the evacuees were transported safely to the appropriate VA facility. The key to managing this task was the partnership skills of USPHS team leadership.

Finally, the surrounding community worked with the evacuees to place pets in animal shelters, obtain oxygen tanks and concentrators from respiratory-supply companies and sample medications from pharmaceutical representatives, and identify ombudsmen among leaders emerging from the evacuee community. 

This assignment demonstrated how three federal assets as well as state and local stakeholders worked together to address the needs of a group of evacuated people that included a large percentage of individuals with chronic diseases. Our experience in this partnership leads to the following recommendations:

1. Assume that evacuees have chronic medical problems requiring assessment. Evacuees who are sheltered but not sorted or triaged and without medical support may rapidly develop complications from decompensating chronic disease and injury.

2. Conduct secondary and tertiary triage soon after primary triage to rapidly identify developing complications.

3. Implement one-on-one clinical triage of evacuees; the process is time consuming but mandatory for good outcomes.

4. Include dedicated behavioral health personnel prepared for acute and chronic intervention and incorporate Critical Incident Stress Debriefing (5) into shelter management.

5. Ensure that the emergency shelter includes a balance of acute and chronic health care personnel and individual-level and population-level health care personnel.

6. Design a PRECEDE–PROCEED-type tool for program planning and evaluation (6) to assist future multidisciplinary healthcare teams in fulfilling an emergency-response assignment. The phases of this system — including social assessment and situational analysis, epidemiologic assessment, educational and ecological assessment, administrative and policy assessment, intervention alignment, implementation, and process and outcome evaluation — could be applied to emergency situations, including national disasters, to help public health practitioners reach their health-related goals.
